# A49 PROSPECTIVE PAIRED COMPARISON STUDY OF 2 FRANSEEN-TIP EUS FNB NEEDLES, FLEXIBLE VS STIFF SHEATH

**DOI:** 10.1093/jcag/gwae059.049

**Published:** 2025-02-10

**Authors:** F Borahmah, D Motomura, R Trasolini

**Affiliations:** Department of Gastroenterology, The University of British Columbia, Vancouver, BC, Canada; Providence Health Care, Vancouver, BC, Canada; Vancouver Coastal Health Authority, Vancouver, BC, Canada

## Abstract

**Background:**

Variety of needle tips and suction techniques have been trialled to optimize diagnostic yield in EUS-FNB for comprehensive histopathological analysis with some studies suggesting improved performance with franseen tip needle

**Aims:**

Obtain an objective comparison of diagnostic yield between 2 different 22g franseen-tip needles; novel flexible sheath (FS) franseen tip needle and traditional stiff sheath (SS) needle

**Methods:**

Sample of patients presenting for outpatient elective EUS-FNB were selected.

Underwent EUS-FNB using both a SS and FS FNB needle during same procedure.

Equal number of passes was obtained with each needle by same endoscopist with random order of sampling, with a planned number of 2 passes per needle; not obtained for one lesion due to difficulty with puncture.

Obtained specimens were sent to pathology; quality of sampling was assessed by the pathologist and cellularity was recorded as a percentage.

In procedures where one needle was unable to puncture or an acellular specimen was obtained a score of 0 was given.

Paired 2 tailed t-test was performed for comparison of mean cellularity. Hazard ratio was calculated for risk of obtaining a non-diagnostic sample

**Results:**

SS mean cellularity 45.7% SD 25.7

FS mean cellularity 72.9% SD 17.0

Difference in mean cellularity 27.2% two-tailed p-value=.08

Hazard ratio of obtaining a non-diagnostic sample with SS: HR 5 p=0.28

No statistically significant difference in results but a numerical trend is noted.

Analysis demonstrates variability in cellularity of specimens obtained with each needle type; 6/8 specimens showed higher cellularity with FS needle while only 1/8 showed higher cellularity with SS.

In 2 cases, both similar lesion types in different patients, non-diagnostic specimens were obtained by SS. One patient due to inability to puncture and in a second due to inadequate cellularity

**Conclusions:**

Results may suggest presence of tissue characteristics or lesion-specific factors that influenced the sampling efficacy.

Findings can provide potential insight into optimizing selection of biopsy needle designs and is a novel look at an uncommonly analyzed aspect of FNB needle design, namely sheath stiffness.

Several limitations of this study, primarily the small sample size

Further research with larger cohorts is needed to compare the diagnostic yields of needle types.

Despite preliminary nature of these results, this study contributes real world paired comparison data

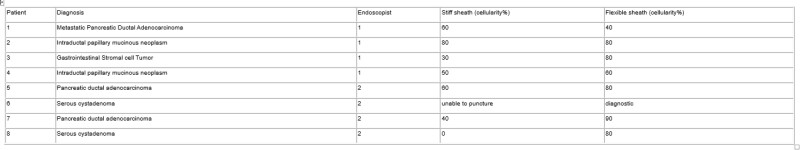

**Funding Agencies:**

